# Predator olfactory cues generate a foraging–predation trade-off through prey apprehension

**DOI:** 10.1098/rsos.150537

**Published:** 2016-02-10

**Authors:** Adam M. Siepielski, Eric Fallon, Kate Boersma

**Affiliations:** Department of Biology, University of San Diego, 5998 Alcala Park, San Diego, CA 92110, USA

**Keywords:** predators, prey, cues, predation risk, olfaction, non-consumptive

## Abstract

Most animals are faced with the challenge of securing food under the risk of predation. This frequently generates a trade-off whereby animals respond to predator cues with reduced movement to avoid predation at the direct cost of reduced foraging success. However, predators may also cause prey to be apprehensive in their foraging activities, which would generate an indirect ‘apprehension cost’. Apprehension arises when a forager redirects attention from foraging tasks to predator detection and incurs a cost from such multi-tasking, because the forager ends up making more mistakes in its foraging tasks as a result. Here, we test this apprehension cost hypothesis and show that damselflies miss a greater proportion of their prey during foraging bouts in response to both olfactory cues produced by conspecifics that have only viewed a fish predator and olfactory cues produced directly by fish. This reduced feeding efficiency is in addition to the stereotypical anti-predator response of reduced activity, which we also observed. These results show that costs associated with anti-predator responses not only arise through behavioural alterations that reduce the risk of predation, but also from the indirect costs of apprehension and multi-tasking that can reduce feeding efficiency under the threat of predation.

## 1. Introduction

Much of the work addressing decision-making in prey has been dedicated to investigating the trade-off between foraging and anti-predator behaviours [1]. While anti-predator behaviours are beneficial because they reduce the risk of predation, missed feeding opportunities can cumulatively lead to marked impacts on reproduction and growth [2–6]. Reduced growth rates associated with anti-predator activities are often thought to arise because organisms reduce the number of foraging bouts [2–4]. This reduction in foraging success is a direct consequence of prey reducing activity levels. However, these costs could also arise through indirect mechanisms.

One potential indirect mechanism yielding anti-predator foraging costs is that cues associated with predator presence may cause prey to be apprehensive in their foraging efforts [7,8]. Apprehension is when a forager redirects attention from foraging tasks to predator detection and incurs a cost from such multi-tasking because the forager ends up making more mistakes in its foraging tasks as a result [7,8]. This would result in an indirect cost associated with foraging under the risk of predation, because it is not generated from prey actively reducing foraging. Such reduced feeding efficiency could generate even greater costs than reduced foraging alone, since energy would be expended for each unsuccessful feeding attempt. While apprehension costs may seem intuitive, few studies have examined the extent to which predators may generate such reduced capture efficiency in their prey [7,8].

Important for generating these anti-predator costs is the ability for prey to detect their predators. Indeed, the presence of multiple types of predator cues in particular may facilitate indirect apprehension costs because prey frequently have multimodal predator detection systems [9–13]. Multimodal predator detection involves the use of visual, tactile, auditory and olfactory cues. Olfactory cues are especially common in aquatic systems where the aqueous environment is conducive to the transmission of chemical signals [9–13]. These chemical cues can be produced directly by predators, by prey that have been injured by predators, and potentially by prey that have been disturbed or stressed (without being injured or consumed) by predators [10,12–14]. The occurrence of the latter disturbance cue remains poorly understood for most animal groups [12]. Similarly, we have little understanding of the general extent to which prey anti-predator responses may differ among the different kinds of predator cues prey use. For example, are prey behavioural responses greater with olfactory cues produced by predators relative to predator detection or disturbance cues produced by conspecifics? Do these cues affect both direct and indirect costs associated with anti-predator activities?

Here we investigate whether both direct (reduced activity levels) and indirect (reduced feeding efficiency) foraging costs to prey can arise through different olfactory predator cues. We use an experimental approach to examine whether larval damselflies, aquatic predatory insects, exhibit shifts in activity levels and foraging efficiency in response to predator detection cues released by conspecifics that have previously been visually exposed to their fish predator, as well as direct olfactory cues from fish predators. We hypothesize that these predator cues will generate costs via both reduced activity levels and increased apprehension. Our findings highlight the importance of often-ignored indirect aspects of behavioural decision-making.

## 2. Material and methods

### 2.1 Study organisms

Damselfly larvae, *Ischnura cervula*, and their fish predators, western mosquitofish (*Gambusia affinis*), were gathered from Water of the Woods pond in the Laguna Mountains of southern California, USA (32°52′46′′ N, 116°27′51′′ W). Damselflies were held in an environmental chamber at 17°C with a 12 L : D cycle and housed individually in 20 ml scintillation vials filled with aged dechlorinated tap water (changed weekly), a wooden perch and fed *Artemia* nauplii every other day. All damselflies molted at least once in captivity prior to experiments. Mosquitofish were held under identical temperature and light conditions in a 37.9 l aquarium and fed *Artemia* and *Daphnia* daily; mosquitofish were not fed damselflies.

### 2.2 Olfactory treatments

To examine whether or not damselflies modify behaviours in response to predator olfactory stimuli, we conducted experiments in which we exposed individual damselflies to water from four treatments: (i) ‘fish’—water containing three mosquitofish, (ii) ‘damselfly with fish’—water previously containing damselflies that observed mosquitofish but had no direct contact with mosquitofish or their chemical cues, (iii) ‘damselfly without fish’—water with damselflies and no fish predator, and (iv) ‘control’—dechlorinated tap water (electronic supplementary material, figure S1). To obtain chemical cues for our treatments, we constructed a clear acrylic container (35×24.5×12.5 cm) filled with dechlorinated tap water (at room temperature—approx. 20°C) that housed fish, with a smaller inner box (10×10.5×12.5 cm) that held a damselfly, but was sealed from the fish arena (electronic supplementary material, figure S1). The inner box had a small piece of fibreglass screen covering the bottom to give damselflies footing. Three mosquitofish (approx. length=6 cm) were placed in the outer container. At the start of each testing day, this container was left undisturbed for 1 h following fish and damselfly additions to allow for the accumulation of chemical signals. The fish treatment water was obtained from water in the outer chamber containing the fish. The damselfly with fish treatment water was obtained from the small inner box where the damselfly could view the mosquitofish but had no direct contact with water containing the predator. An identical container was used to obtain the damselfly without fish treatment, with the exception that fish were absent (electronic supplementary material, figure S1). No *Artemia*or *Daphnia*were fed to fish or damselflies during these experiments. Water from these conditioning periods was then used as treatments in the damselfly behavioural assays. To eliminate cross-contamination, containers were assigned to the same treatment for the duration of the study.

### 2.3 Behavioural assays

Behavioural assays were conducted in a plastic container (9×9×8 cm) filled with 150 ml of dechlorinated tap water at room temperature (approx. 20°C) and a wooden dowel glued to the bottom as a perch. During trials, individual damselflies were placed in the container with approximately 60–70 *Artemia* prey. Damselflies were deprived of food 48 h prior to experiments to ensure motivation for feeding. The day before experimentation damselflies were removed from the environmental chamber and placed in the room where the observations were conducted to acclimate the damselflies to the temperature of the room (approx. 20°C) during the experimental trials.

Individual damselflies were randomly assigned to olfactory treatments and each treatment was replicated 20 times. For logistical reasons, experiments involving the damselfly without fish treatment were conducted with newly sampled individuals two months after the other experiments were completed. Although this meant that some larvae may have been larger than those used in the other treatments, and damselfly larvae behavioural responses to predators can differ among larvae instars (e.g. [3]), individuals in all four treatments generally overlapped in size because *Ischnura*are multivoltine in the study area, so any possible age differences in behaviour were likely minimal and should not confound our comparisons.

Individual behavioural assays were conducted using a paired approach consisting of two 5 min observation periods (modified from [15]). Behaviours were recorded by a single observer over the first 5 min in the absence of any olfactory treatments. During the subsequent 5 min, water treatments (as described above; electronic supplementary material, figure S1) were applied by gently replacing 10 ml of the container water with 10 ml of treatment water, and behavioural data were collected again. We scored behaviours related to the foraging and activity levels of these insects: number of walks, number of swims, number of body moves/bends, number of strikes at prey and the number of prey consumed after a strike (see [6,16,17] for details). We also calculated the proportion of prey consumed to prey strikes as a measure of foraging efficiency. While each of these different behaviours are not independent, our goal was not to determine what, if any, specific behaviours drive the patterns we found. Rather, we simply wanted to examine how damselflies may respond to different olfactory cues by altering behaviours, and to be able to make direct comparisons with previous studies (e.g. [15,16]).

To examine whether olfactory cues modify damselfly behaviours and foraging efficiency, we calculated the difference in each behaviour before and after treatment application. We then used these differences as the response variable in a Kruskal–Wallis test, because although variances were equal based on Levene's tests (all *p*>0.05), the data were non-normally distributed (Shapiro–Wilk test). *Post hoc* pairwise comparisons (at *α*=0.05) were implemented in the *pgirmess* package [18] of program R (R Core Team 2014).

## 3. Results

**Figure 1.**
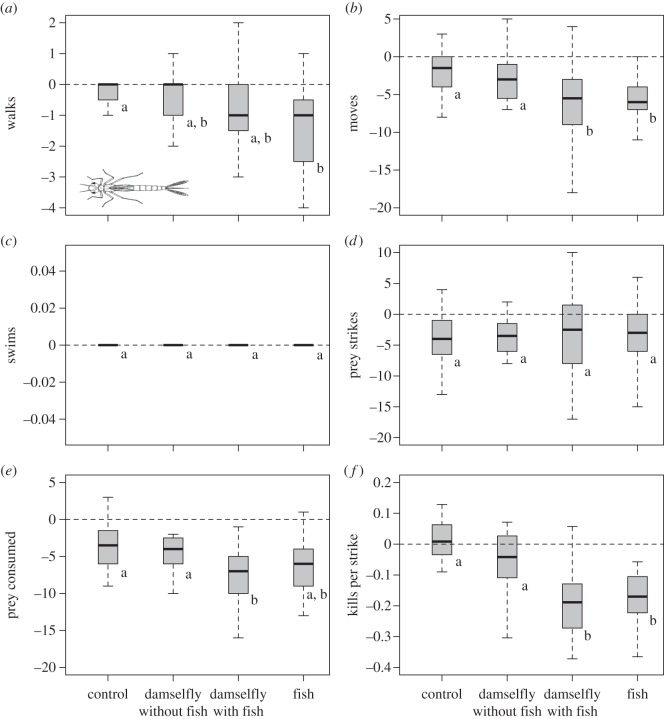
Damselflies reduce activity and become less efficient predators in the presence of predator olfactory cues. Shown are boxplots (line is the median, box is interquartile distance (IQD) and the whiskers denote ±1.58IQD) of the difference in a given damselfly behaviour before and after the application of predator cue treatments (*n*=20 individuals per treatment). Statistically significant differences (*p*<0.05) between treatments are denoted by the absence of shared letters. Image credit: CSIRO Australian National Insect Collection. Data from the experiment are deposited in the Dryad Digital Repository [19].

Predator cues reduced the number of walks among treatments (Kruskal–Wallis *χ*^2^=12.86, d.f.=3, *p*=0.005), but only when comparing the fish and control treatments (figure 1*a*). The number of moves also decreased in comparisons involving predator cues (Kruskal–Wallis *χ*^2^=13.90, d.f.=3, *p*=0.003), both comparing the damselfly with fish detection and fish treatments to the control and damselfly no fish treatments (figure 1*b*). The number of swims did not differ significantly among treatments (Kruskal–Wallis *χ*^2^=1.62, d.f.=3, *p*=0.654; figure 1*c*), although swims were rarely observed.

The number of strikes (Kruskal–Wallis *χ*^2^=0.81, d.f.=3, *p*=0.847) did not differ significantly among treatments (figure 1*d*); however, the number of prey items successfully consumed during these strikes did (Kruskal–Wallis *χ*^2^=11.43, d.f.=3, *p*=0.009). We are uncertain why the number of strikes uniformly declined after treatments were added; it could reflect damselflies becoming satiated prior to exposure to the treatments, prey depletion leading to lower prey densities and thus prey encounter rates, or a possible response to the addition of the treatments that for some reason was not exhibited in the other behaviours. Interestingly, only the comparison between the damselfly with fish and the control treatment led to a significant reduction in the number of prey consumed (figure 1*e*). The number of prey consumed relative to the number of strikes (efficiency) also varied across treatments (Kruskal–Wallis *χ*^2^=31.83, d.f.=3, *p*<0.0001) with an almost 20% reduction in the proportion of successful attacks when either fish or damselfly with fish detection cues were present relative to the control or damselfly no fish treatments (figure 1*f*).

## 4. Discussion

Consistent with the apprehension hypothesis we found that damselflies exhibited reduced foraging efficiency under the threat of predation because they were less successful at capturing their own prey. This indirect foraging cost is in addition to the direct costs of reduced activity levels. The magnitudes of these foraging costs were similar for cues produced by fish as for disturbance cues produced by conspecifics. Below we argue for the importance of olfactory cues in facilitating these apprehension costs and therefore shaping the foraging/anti-predator trade-off.

We found that damselflies exhibited anti-predator behaviours in response to olfactory cues produced by conspecifics that have observed a predator, as well as to direct olfactory cues released from the predators themselves. In combination with previous studies, these results show that damselflies can detect and respond to predation risk through at least four classes of olfactory cues: cues from predators [16], cues from conspecifics responding to the presence of predators (this study), cues from damaged conspecifics [15,16] and cues given off by heterospecifics [16]. The responses we observed are also comparable to damselfly responses to visual predator cues [6].

Studies examining olfactory cues given off directly from predators often argue that prey respond to cues given off by predators because they have consumed conspecifics or heterospecifics, rather than cues produced directly by predators in the absence of predation [10,15,16]. In our experiment, fish were not fed damselflies during the study. Thus, fish cues were most likely from the fish themselves. It is possible that damselflies could have responded to chemical cues from fish that had previously fed on *Daphnia* or *Artemia*prior to the experiments. However, any such behavioural responses would seem costly as these are the same prey that damselflies consume, and odonates use olfaction to detect prey [20]. Our results also show that damselflies release a predator detection chemical cue into the environment and that conspecifics readily respond to this cue, which may belong to a class of ‘disturbance’ cues [10–13,21]. This suggests the possibility that cues from conspecifics being consumed by predators [15] may actually arise from the release of a predator detection chemical rather than injured conspecifics *per se* (e.g. [14]). While we do not know the chemical nature of this cue, some have suggested that this kind of disturbance cue may be an ammonia-based metabolic by-product [22–24]. Importantly, the lack of any anti-predator response in the treatment consisting of water from a damselfly without any predator exposure verifies that the response was not simply due to conspecifics. The latter finding also implies that any possible non-predator olfactory cues given off by damselflies would likely not generate interference competition costs (i.e. reduced foraging success because of intraspecific competitor presence [25]). Thus, these chemical signals seem to be reliable indicators of the presence of predators.

While some studies have found that olfactory cues released by conspecifics or heterospecifics elicit less of a response than cues produced directly by predators and may primarily act to alert prey to the presence of predators [12], our results show that the magnitudes of damselfly responses to both fish and damselfly conspecific detection cues were comparable. These responses may vary more under more complex natural habitats. Our experimental set-up lacked any cover for damselflies and so damselfly responses may have been more extreme because perceived risk was exaggerated [26]. Additionally, responses to these signals may be additive such that a combined fish and damselfly conspecific cue together could illicit an even greater response [27]. Regardless, in all cases these cues led to reductions in activity levels that should reduce the risk of predation by fish [28]. Indeed, this pattern is found in many studies examining how damselflies and other animals respond to the threat of predation [3,6,29–31].

Although we did not quantify the growth costs associated with reduced activity levels, other research on damselflies suggests it is likely that reduced activity would mean that individuals stay in low prey density locations longer and fail to locate higher prey density locations [31]. Damselflies are food limited [32], so reductions in food capture reduce growth rates [3,30,31]. These reductions in activity levels reflect the direct costs of changing activity that generate the growth–predation trade-off [1].

While these direct costs of reduced foraging activity are widely observed, we also found that damselflies under risk of predation exhibited a reduction in feeding efficiency. In our experiment, damselflies showed no changes in the number of feeding strikes among treatments. However, when predator cues were present we observed a marked decline of almost 20% in their success at capturing their prey once an attack was initiated. That is, damselflies were less lethal under the threat of predation. This resulted in an indirect cost associated with foraging under the risk of predation because it does not stem from damselflies actively reducing activity levels. It seems unlikely that damselflies would intentionally choose to miss a potential prey item. Attacks require energy to perform and the movement associated with them could increase detectability to a predator. There should thus be a premium on successful feeding attempts. This may especially be the case for sit-and-wait predators like damselflies that do not actively pursue prey.

We hypothesize that the reduction in successful prey attacks is because predator cues cause damselflies to become apprehensive and redirect attention from foraging tasks to predator detection [7,8]. The ability to engage successfully in multiple concurrent activities is limited and this limit can affect both foraging and vigilance [7,8,33,34]. In general, odonates are thought to be efficient predators because they have exquisite vision and a nervous system that is well developed for capturing prey [35], allowing them to accurately estimate the distance to potential prey and exhibit high capture efficiency [36]. Odonates can also use olfaction to detect prey [20]. Despite these complementary sensory systems, we found that their success rate while attacking prey declined when presented with two forms of predator olfactory cues (those released by fish and those released by conspecifics). We suspect that the apprehension created by these predator presence cues caused damselflies to hesitate and decrease their accuracy in targeting prey.

One alternative explanation for reduced feeding efficiency of damselflies in the presence of the fish olfactory cue is that this cue may have caused the damselfly prey, *Artemia*, to become more alert and thus reduce damselfly foraging success. Although we saw no obvious changes in *Artemia* behaviours among treatments, at least over the short duration of our study, some studies have suggested that *Artemia* may swarm in response to the threat of fish predation [37]. If *Artemia* were swarming and doing so away from where damselflies were perched, this could result in a reduction in damselfly feeding efficiency over the short run. Unfortunately, we did not record data on *Artemia* locations relative to damselfly locations. Future studies would benefit from further investigating this possibility.

In summary, these results support the idea that foraging under the threat of predation entails costs. However, we highlight two often overlooked aspects of the foraging/anti-predator behaviour trade-off. First, anti-predator behaviours may arise through predator olfactory cues or through cues given off by disturbed conspecifics that have encountered a predator, even if this encounter was purely visual. Second, predator detection by a prey species may impact prey fitness by reducing the prey species own capture efficiency once a feeding attempt has been initiated. This latter indirect apprehension cost may be a prominent aspect of the foraging ecology of many organisms and play an important role in shaping the foraging/anti-predator trade-off [7,8].

## Supplementary Material

Figure S1

## Supplementary Material

Siepielski et al predator cue data

## Data accessibility

All data are available on Dryad (doi:10.5061/dryad.4p57t).
