# Supplementary material for: Predator olfactory cues generate a foraging–predation trade-off through prey apprehension
Source: R Soc Open Sci. 2016 Feb 10;3(2):150537. doi: 10.1098/rsos.150537 (PMC4785975; doi:10.1098/rsos.150537)
Supplement: Figure S1 [file rsos150537supp1.pdf]

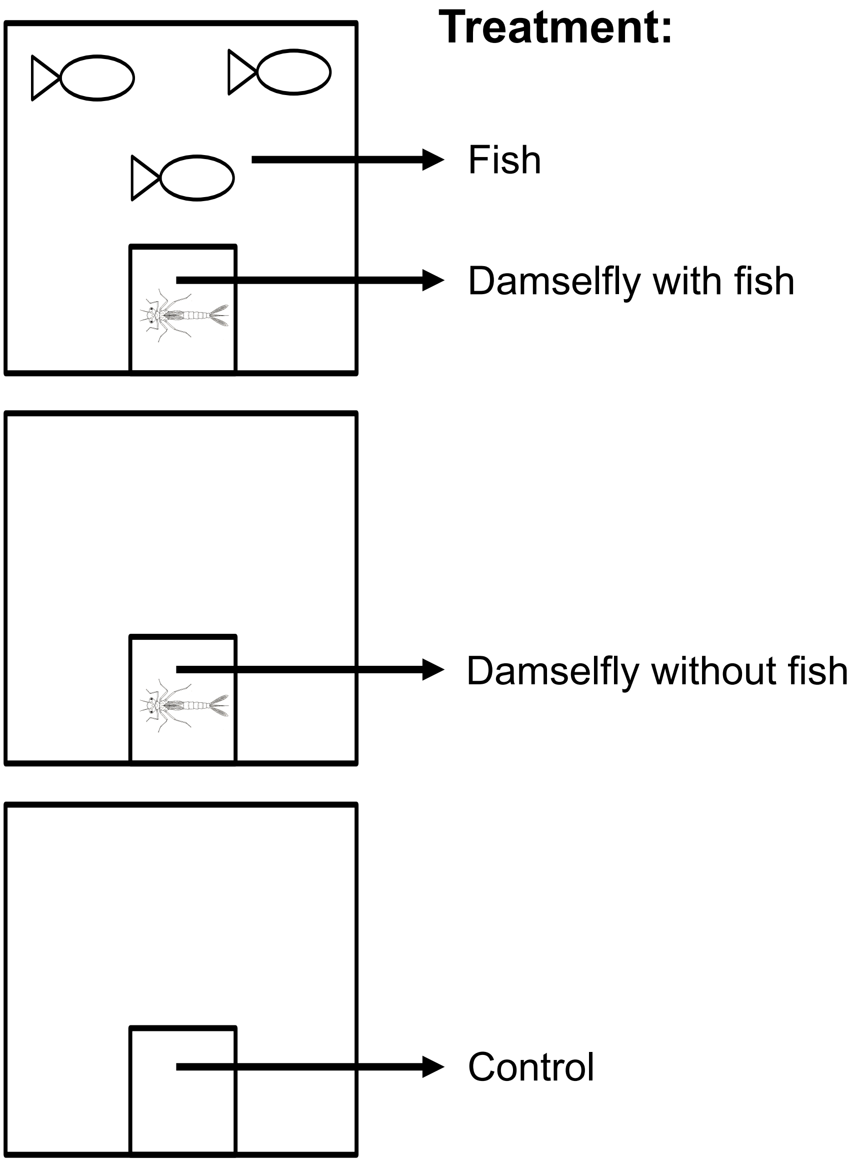

Figure S1. Schematic illustrating the sources of water used in generating the olfactory treatments. The schematic depicts the experimental set-up as viewed from above.
